# Preceding anti-spike IgG levels predicted risk and severity of COVID-19 during the Omicron-dominant wave in Santa Fe city, Argentina

**DOI:** 10.1017/S0950268822001716

**Published:** 2022-11-03

**Authors:** Ayelen T. Eberhardt, Melina Simoncini, Carlos Piña, Germán Galoppo, Virginia Parachú-Marco, Andrea Racca, Sofía Arce, Evangelina Viotto, Florencia Facelli, Florencia Valli, Cecilia Botto, Leonardo Scarpa, Celina Junges, Cintia Palavecino, Camila Beccaria, Diego Sklar, Graciela Mingo, Alicia Genolet, Mónica Muñoz de Toro, Hugo Aimar, Verónica Marignac, Juan Carlos Bossio, Gustavo Armando, Hugo Fernández, Pablo M. Beldomenico

**Affiliations:** 1 Laboratorio de Ecología de Enfermedades, Instituto de Ciencias Veterinarias del Litoral (ICIVET-Litoral), Universidad Nacional del Litoral – Consejo Nacional de Investigaciones Científicas y Técnicas (UNL-CONICET), Esperanza, Argentina; 2 Centro de Investigación Científica y de Transferencia Tecnológica a la Producción-Consejo Nacional de Investigaciones Científicas y Técnicas-Provincia de Entre Ríos-Universidad Autónoma de Entre Ríos, Diamante, Argentina; 3 Facultad de Ciencia y Tecnología, Universidad Autónoma de Entre Ríos, Diamante, Entre Ríos, Argentina; 4 Laboratorio de Ecofisiopatología – Instituto de Salud y Ambiente del Litoral (ISAL) Universidad Nacional del Litoral – Consejo Nacional de Investigaciones Científicas y Técnicas (UNL-CONICET), Santa Fe, Argentina; 5 Facultad de Bioquímica y Ciencias Biológicas, Universidad Nacional del Litoral (FBCB-UNL), Santa Fe, Argentina; 6 Laboratorio de Ecología Molecular Aplicada, Instituto de Ciencias Veterinarias del Litoral-Universidad Nacional del Litoral – Consejo Nacional de Investigaciones Científicas y Técnicas (UNL-CONICET), Esperanza, Argentina; 7 Facultad de Ciencias Veterinarias, Universidad Nacional del Litoral, Esperanza, Santa Fe, Argentina; 8 Laboratorio de Biología Celular y Molecular Aplicada, Instituto de Ciencias Veterinarias Del Litoral (ICIVET-Litoral), Universidad Nacional Del Litoral (UNL), Consejo Nacional de Investigaciones Científicas y Tecnológicas (CONICET), Esperanza, Santa Fe, Argentina; 9 Instituto de Matemáticas Aplicadas del Litoral (IMAL), Universidad Nacional del Litoral – Consejo Nacional de Investigaciones Científicas y Técnicas (UNL-CONICET), Santa Fe, Argentina; 10 Instituto de Estudios Sociales (INES), Universidad Nacional de Entre Ríos-Consejo Nacional de Investigaciones Científicas y Técnicas (UNER-CONICET), Paraná, Argentina; 11 Laboratorio de Investigación en Enfermedades Infecciosas, Dr Néstor Bianchi, Hospital San José de Diamante, Entre Ríos, Argentina; 12 Instituto Nacional de Enfermedades Respiratorias ‘Dr Emilio Coni’, Santa Fe, Argentina

**Keywords:** Antibody titre, disease severity, humoral defences, infection risk, longitudinal study, pre-exposure, SARS-CoV-2

## Abstract

The SARS-CoV-2 Omicron variant has increased infectivity and immune escape compared with previous variants, and caused the surge of massive COVID-19 waves globally. Despite a vast majority (~90%) of the population of Santa Fe city, Argentina had been vaccinated and/or had been infected by SARS-CoV-2 when Omicron emerged, the epidemic wave that followed its arrival was by far the largest one experienced in the city. A serosurvey conducted prior to the arrival of Omicron allowed to assess the acquired humoral defences preceding the wave and to conduct a longitudinal study to provide individual-level real-world data linking antibody levels and protection against COVID-19 during the wave. A very large proportion of 1455 sampled individuals had immunological memory against COVID-19 at the arrival of Omicron (almost 90%), and about half (48.9%) had high anti-spike immunoglobulin G levels (>200 UI/ml). However, the antibody titres varied greatly among the participants, and such variability depended mainly on the vaccine platform received, on having had COVID-19 previously and on the number of days elapsed since last antigen exposure (vaccine shot or natural infection). A follow-up of 514 participants provided real-world evidence of antibody-mediated protection against COVID-19 during a period of high risk of exposure to an immune-escaping highly transmissible variant. Pre-wave antibody titres were strongly negatively associated with COVID-19 incidence and severity of symptoms during the wave. Also, receiving a vaccine shot during the follow-up period reduced the COVID-19 risk drastically (15-fold). These results highlight the importance of maintaining high defences through vaccination at times of high risk of exposure to immune-escaping variants.

## Introduction

As of September 2022, the COVID-19 pandemic continues to occur despite the acquired defences developed in a large proportion of people due to vaccination and/or natural infection by SARS-CoV-2. Several viral variants have evolved, prevailing the ones that achieved enhanced transmissibility and immune escape compared to prior variants [1]. Until November 2021, some strains had become prominent and had caused new outbreaks worldwide. These were considered variants of concern, and were named Alpha, Beta, Gamma and Delta. A new variant, B.1.1.529 was first detected in samples collected on 11th November 2021 in Botswana and on 14th November 2021 in South Africa [1]. On 26th November, the WHO defined it as the fifth variant of concern, naming it Omicron. So far, Omicron is the variant with the largest number of mutations, many of which provide increased infectivity and immune escape compared with previous variants [2, 3]. This resulted in massive waves of COVID-19 emerging worldwide soon after the new variant appeared [4].

The dynamics of COVID-19 have been heterogeneous since the beginning of the pandemic [5]. While countries like United Kingdom and Germany have gone through several epidemic waves, others like Thailand and Vietnam had their first wave only after over a year of relatively silent viral circulation. In Argentina, by early December 2021 there had been two waves, the first one by mid-2020, related to the arrival and spread of the virus, and the second one in 2021 associated with the seasonality of respiratory viruses. Omicron was confirmed in Argentina on 5th December 2021, and some days later the country endured the largest COVID-19 epidemic wave so far, with a peak infection rate several times higher than the peaks observed in the two previous waves.

**Fig. 1.**
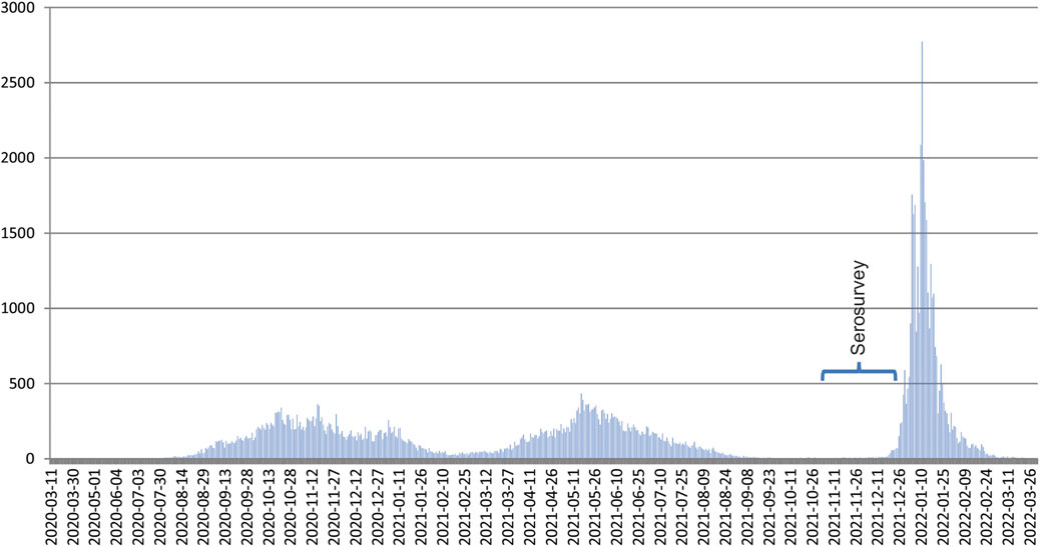
Temporal distribution of the confirmed cases of COVID-19 in Santa Fe city (official records of the Ministry of Health of Santa Fe province).

In Santa Fe, a city of around 430 000 inhabitants, COVID-19 dynamics reflected what was observed elsewhere in Argentina (Fig. 1). By mid-December 2021, 12.9% of the citizens had been diagnosed with COVID-19, 90.6% had received a first dose of an anti-SARS-CoV-2 vaccine, 79.2% a second dose and 10.3% a third one (data provided by the Ministry of Health of Santa Fe province). The wave that followed the arrival of Omicron began around 18th December 2022 in Santa Fe city, and the number of daily cases started to decline by mid-January 2022, returning to levels as low as before the wave by the end of February (Fig. 1).

During November and December of 2021, we conducted a survey collecting relevant information on COVID-19 and measuring anti-spike immunoglobulin G (IgG) antibodies in people from randomly selected households of Santa Fe city and from citizens that volunteered to participate in the study. This provided the opportunity of characterising the acquired humoral defences of the population of Santa Fe city immediately prior to the arrival of Omicron. In March 2022, after the wave was over, a subset of the study participants was asked to complete a second questionnaire indicating if they were diagnosed with COVID-19 after 15th December 2021, if they got additional vaccine shots, and other relevant information. The data collected allowed us to pursue three goals:

1. to describe the acquired humoral immunity of the population immediately prior to the arrival of Omicron,
2. to assess which factors were associated with such immune status (i.e. previous infection, different vaccination schemes, time from last exposure, etc.) and
3. to evaluate if those humoral defences predicted the risk and severity of COVID-19 during the wave.

## Materials and methods

### Source of the data

A random sample of 1000 households including all neighbourhoods of Santa Fe city was provided by the Instituto Provincial de Estadísticas y Censos. In September and October 2021, those households were visited, and the occupants were invited to participate in a COVID-19 study that involved answering a questionnaire and providing a blood sample to measure IgG antibodies against SARS-CoV-2. A second visit was scheduled from 1st November 2021 to 23rd December 2021, to fill the questionnaires and take a blood sample. In addition, volunteers were invited to participate by announces in the local media. We collected data from 414 people from randomly selected households and 1041 volunteers.

The first questionnaire included queries on sex, age, having been diagnosed with COVID-19 (with dates and diagnosis details), COVID-19 severity and duration, vaccine shots received (with type and dates), close contacts with COVID-19 cases, co-morbidities, among other information.

Those that were sampled after 15th November 2021 were asked to complete a second questionnaire in March 2022. This allowed us to follow the participants from whom there was an antibody measurement within a month prior to the Omicron-dominant wave, which gave us confidence that the antibody levels recorded reflected the humoral defences with which each participant faced the wave. The second questionnaire inquired for the period that went from the date of the blood sample collection to 28th February 2022, and included information on close contact with cases during that period, COVID-19 diagnosis (with dates and diagnosis details), vaccine shots (with type and dates) and disease severity and duration. When there were doubts about the responses given in the questionnaires, the participants were contacted again asking for clarification. Of the 843 participants that were invited to answer the second questionnaire, 514 provided valid responses.

Using the information obtained from the questionnaires, we established a COVID-19 case when the participant indicated that she/he was given positive by the government after an official polymerase chain reaction (PCR) test, or given positive by the government due to having symptoms while cohabitating with a case, or had a PCR positive test by a private laboratory, or had a positive rapid antigen test while having symptoms or after having had a close contact with a case. People that declared they suspected having had COVID-19 but were not tested nor considered positive by the government (25 in the first questionnaire and four in the second questionnaire) were removed from the analysis.

All procedures were carried out under the approval of the Ethics and Biosafety Committee of the Scientific and Technological Centre of Santa Fe of the Argentine Council for Research and Technology (CCT Santa Fe CONICET). All participants signed an informed consent form.

### Quantification of IgG

Levels of anti-SARS-CoV-2 spike protein IgG were quantified by COVID AR IgG immunoassay developed by Instituto Leloir in Argentina [6], following the manufacturer's instructions. This IgG immunoassay kit consists of a solid phase enzyme-linked immunoassay that utilises as antigens the trimer of native protein S and a domain of that SPIKE protein that contains the receptor binding domain, obtained by recombinant DNA techniques produced in human cells.

Briefly, 40 μl of fingertip capillary blood samples were diluted 1:6 in the diluent provided in the SEROKIT developed by Instituto Leloir, and kept refrigerated. At the laboratory, samples were re-diluted 1:3, and 200 μl of each 1:18 final dilution were transferred to 96-well plates and incubated at 37°C for 1 h. IgG specific for spike protein was captured on the plate, and subsequently the wells were thoroughly washed six times to remove unbound material. Anti-human IgG, horseradish peroxidase (HRP)-linked antibody was then used to recognise the bound IgG. A mix of HRP substrate and TMB (1:1) was added to develop colour. The magnitude of optical density at 450 nm is proportional to the quantity of IgG specific for spike protein. To estimate antibody levels, sample optical densities were converted to concentrations expressed in UI/ml by using a lineal model built with the optical densities (response variables) obtained in each plate from two sets of known dilutions of the positive control at 50, 100, 200 and 400 UI/ml. These dilutions were the independent variable, included as a polynomial term (with lineal and quadratic terms) to address possible non-linearity of the dilution–OD (optical density) relationship. The *R*^2^ of that model was checked to confirm that the value was >0.85. When the value was below that threshold all samples were analysed again in a new plate.

### Statistical analysis

All statistical analyses were done using software R version 4.2.0 (The R Foundation for Statistical Computing). The analyses were conducted in three steps, to pursue three complementary goals, as follows.

The first step aimed to characterise the acquired humoral defences in Santa Fe immediately prior to the arrival of Omicron. This part consisted of descriptive statistics of the IgG levels, overall and by age group, using data from the 1455 participants that answered the first questionnaire and provided a blood sample.

The goal of the second step was to investigate the determinants of the IgG levels measured, using the same dataset as for the first step, but eight participants were removed from this analysis because they had received the last vaccine shot briefly before the blood sample (up to 7 days). For this step, the antibody levels were the response variable, which were transformed by calculating the square root to approach normality. Two sets of lineal models were run, one containing the number of doses as variable of interest (levels = 0, 1, 2 and ≥3), and the second set including only the four vaccination schemes most frequently observed, to compare antibody levels among them. In both models, the independent variables ‘COVID-19’ (prior diagnosis of COVID-19) and ‘days from last exposure’ (vaccine shot or detected infection; whatever happened last), were included. The vaccination schemes used for the second model were: two Astra-Zeneca vaccines (viral vector vaccine; *N* = 411), two Sinopharm (inactivated vaccine; *N* = 334), two Sputnik V vaccines (viral vector vaccine; *N* = 260), the combination of Sputnik V and Moderna (viral vector + mRNA vaccines; *N* = 155) and two Pfizer/BioNTech (mRNA vaccine; *N* = 25).

The third step used information from the second questionnaire (*N* = 514) to conduct a longitudinal analysis that enabled assessment of how the vaccines and antibody levels influenced the incidence of COVID-19 during the Omicron-dominant wave in Santa Fe. The subset of data used for this third step (*N* = 484) excluded participants that got a vaccine shot between 7 days prior of the blood sample collection and 15th December 2021 (*N* = 26) and individuals that declared they suspected having had COVID-19 during the wave but were not tested (*N* = 4). In addition, we looked at associations between antibody levels and COVID-19 symptoms severity and duration among those that were infected during the Omicron-dominant wave (*N* = 174). For this third step, the period in which the participants were followed-up to assess new detected infections by SARS-CoV-2 was from 18th December to 28th February (72 days).

In order to establish an association between vaccination status and the incidence of COVID-19 during the Omicron-dominant wave, we built a generalised lineal model (GLM) with a binomial response (COVID-19 positive or not). The model used the number of vaccine doses as the independent variable of interest (levels = 0, 1, 2 and ≥3).

To assess associations between antibody levels and the incidence of COVID-19 during the Omicron-dominant wave, we built a GLM with a binary response, in which the independent variable of interest were the IgG levels, and a number of other variables (detailed below) were included to control for potential confounding phenomena. The independent variable of interest was also included in a separate model as a dichotomous factor, setting those with antibody levels >400 UI/ml (very high levels; *N* = 158) as 1, and the rest as 0 (*N* = 326).

Finally, in the subset of samples that was diagnosed with COVID-19 during the wave (*N* = 174) the associations between antibody levels and COVID-19 severity and duration were assessed with ordinal regression models, where the responses were three-level ordinal variables, as follows. Disease severity was measured by asking in the second questionnaire whether they had no or very mild symptoms (e.g. light sore throat, nasal congestion; level 1), mild symptoms (e.g. 1 or 2 days of fever and/or light malaise, not requiring bed rest; level 2) or moderate symptoms (e.g. bed rest was required; level 3). The participants were also asked if hospitalisation was required, as a 4th level, but none chose this option. As for the duration of COVID-19 symptoms (excluding loss of smell), the three levels were: 1 day or less (level 1), 2–5 days (level 2) and more than 5 days (level 3).

For all models used in step 3, potential confounding phenomena was controlled for by including in the models relevant independent variables, as follows. Age (in years, and assessed separately as a single term or polynomial) was included in all models. Also in all models, receiving a new vaccine during the wave period was included as a two-level independent variable, as those that got a booster shot within the follow-up period had changes in both the antibody levels and the vaccination scheme. The cases in which the vaccine shot was received late in the wave (after 15th February 2022; *N* = 7) were excluded in the models that included the new shot variable. The number of known close contacts with COVID-19 cases was used as a proxy of exposure, and included for adjustment in the GLMs assessing associations with COVID-19 incidence. Close contact was defined as being within 3 m distance or indoors for over 15 min with someone who was diagnosed with COVID-19, and the contact happened within the period that went from 2 days prior to the onset of symptoms and 7 days after the onset of symptoms. The contacts were set at 5 levels, 0, 1, 2, 3 and 4 or more close contact with cases. Prior COVID-19 was included in models that assessed associations between vaccination status and COVID-19 incidence during the wave. Finally, the presence of co-morbidities (i.e. high blood pressure, diabetes, obesity, heart disease, chronic pulmonary disease, cancer) was included in the models assessing the influence of antibody levels on COVID-19 severity and duration. All these variables used for adjustment purposes were dropped from the models if they were not important for the model's goodness of fit, as indicated by Akaike's information criterion (AIC) [7]. When the inclusion of a given term did not reduce AIC values in two or more units, the term was dropped from the model. *Post hoc* multiple comparisons of independent variables with three or more levels were done by Tukey's HSD tests, using the function *glht* of the *multcomp* package in R.

## Results

### Description of the sample

We obtained answers to the first questionnaire and blood samples from 1455 people from all neighbourhoods of Santa Fe city. Of those, 57.3% were female and 43.7% were male. The mean age was 41 years, the minimum being 5 months and the maximum 95 years.

Almost three quarters (1076/1455) of the participants had not been diagnosed with COVID-19 at the time of answering the first questionnaire, but 2.3% of those (*N* = 25/1076) suspected having been infected. One quarter (357/1455) was diagnosed with COVID-19 once, and 0.4% (*N* = 6/1455) twice.

Regarding the vaccination regime, 6.9% (100/1455) of the participants were not vaccinated at the time of sampling, 5.0% (73/1455) had one dose, 83.6% (1216/1455) had two doses, 4.5% (65/1455) had three doses and 0.07% (10/1455) had four doses. The vaccination scheme most frequently applied in the sample was two Astra-Zeneca vaccines (*N* = 411), followed by two Sinopharm (*N* = 334), two Sputnik V vaccines (*N* = 260) and the combination of Sputnik V and Moderna (*N* = 155). At the time of the sampling, Pfizer/BioNTech vaccines were being used for youngsters aged 13–18 years, having 1.7% (25/1455) of the participants of our study two doses, and 1.3% (19/1455) one dose.

### Characterisation of the acquired humoral defences prior to the Omicron-dominant wave

Anti-spike IgG were detected in 88.7% (1290/1455) of the samples. Among those that received at least one dose of an anti-COVID-19 vaccine, 7.4% (100/1354) did not have detectable IgG. Among the non-vaccinated (6.8%; 99/1455), 63.6% (63/99) did not have detectable antibodies. Of the unvaccinated that had antibodies, 72% (26/36) had not been diagnosed with COVID-19 nor suspected having been infected.

Almost half (48.9%; 707/1455) of the participants had antibody levels considered to be high (>200 UI/ml), and more than one-third (35.4%; 515/1455) had very high levels (>400 UI/ml). The overall mean antibody level was 290 UI/ml, but it varied by age group (Table 1). Among the age group considered to be of high risk (>60 years old), the vast majority was vaccinated (98.1%; 305/311), but 17.0% (53/311) was vulnerable because they had no detectable IgG (6.1%; 19/311) or had low antibody levels (10.9% (34/311) with <40 UI/ml). However, most aged 60 and above had very high antibody levels (65.0% (202/311) with >400 UI/ml). The high level of antibodies observed in those aged 13–20 is attributable to the good performance of the vaccine received by that age group and the shorter time elapsed from the last shot (youngsters were vaccinated after older people).

**Table 1.** Central tendency (mean and median) of antibody levels and proportion of vaccine coverage (at least one shot) by age group, in samples taken from Santa Fe citizens in November and December 2021

Age range (years)	Sample size	Mean; median (UI/ml)	% without antibodies	% vaccinated
**0–12**	84	175; 12	43.4	47.0
**13–20**	87	474; 499	9.0	85.4
**21–40**	535	217; 82	12.2	95.8
**41–60**	438	307; 223	9.5	96.0
**>60**	311	378; 408	5.9	98.1

### Antibody levels according to vaccine doses and schemes

**Fig. 2.**
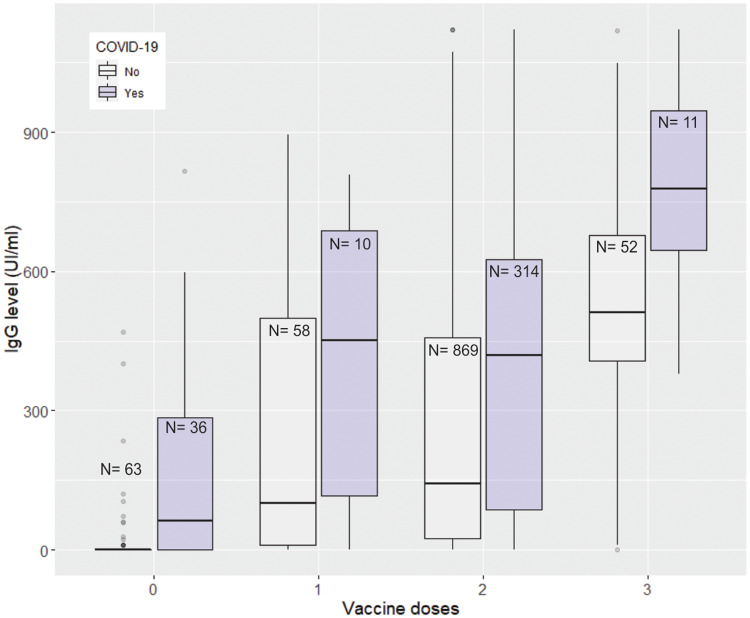
Levels of antibodies (IgG) against SARS-CoV-2 by the number of vaccine doses and prior COVID-19 diagnosis.

Those participants that were not vaccinated had a mean IgG titre of 62.5 UI/ml, while the mean level was 287.9, 293.5 and 567.8 UI/ml for those who received one, two or three doses, respectively. A third dose increased the antibody levels significantly. There was strong positive association between antibody levels and prior COVID-19 and a strong negative correlation with days that elapsed from last exposure (vaccine or infection) (Fig. 2, Table 2).

**Table 2.** Determinants of antibody levels prior to the Omicron wave in Santa Fe city

Model = Antibody levels^0.5^ ~ Vaccine doses + COVID-19 + Days from exposure (*N* = 1447)			
Term	Coefficients	Standard error	*P*-value
Intercept	14.9358	1.914628	<0.0001
*Vaccine doses (1)*	0.518339	2.101330	0.8052
*Vaccine doses (2)*	2.2971	1.7247	0.1831
***Vaccine doses (≥3)***	**8.0837**	**2**.**1069**	**0**.**0001**
**COVID-19**	**4**.**9191**	**0**.**5302**	**<0**.**0001**
**Days from exposure**	**−0**.**0378**	**0**.**0038**	**<0**.**0001**

Lineal model assessing the association between antibody (IgG) levels and number of anti-COVID-19 vaccine doses received, adjusting by prior COVID-19 diagnosis and days from last exposure (vaccine or known infection). Significant terms are printed in bold and the independent variables of interest are italicised.

Antibody levels: titre of anti-SARS-CoV-2 spike protein IgG.

Vaccine doses: number of vaccine shots received prior to the blood sample (4 level variable: 0, 1, 2 and ≥3; reference value = 0).

COVID-19: prior diagnosis of COVID-19.

Days from exposure: days elapsed from the last vaccine shot or detected infection.

**Fig. 3.**
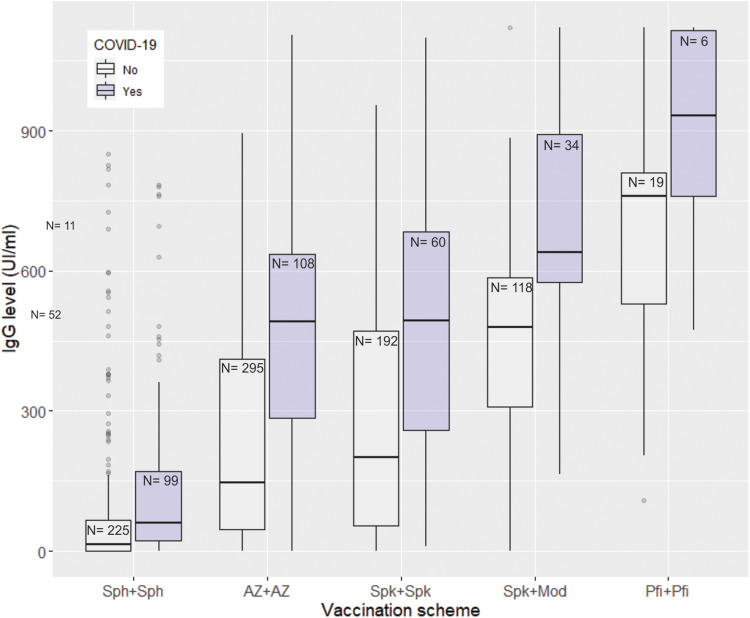
Levels of antibodies (IgG) against SARS-CoV-2 by vaccine scheme and prior COVID-19 diagnosis. Sph, Sinopharm; AZ, Astra Zeneca; Spk, Sputnik V; Mod, Moderna; Pfi, Pfizer/BioNTech.

When comparing the most frequently used vaccination schemes (while adjusting by prior COVID-19 infection and days elapsed from last exposure), we observed very significant differences in antibody levels (Table 3; Fig. 3). The scheme with inactivated vaccines (Sinopharm) showed significantly lower antibody levels than all other schemes, both schemes of viral vectors (Astra-Zeneca and Sputnik V) performed similarly, and the schemes combining vector and mRNA (Sputnik V + Moderna) and two mRNA (Pfizer/BioNTech) showed the highest levels, not statistically different between them (Table 3).

**Table 3.** Lineal model assessing the association between antibody (IgG) levels and different anti-COVID-19 schemes, adjusting by prior COVID-19 diagnosis and days from last exposure (vaccine or known infection)

Model = Antibody levels^0.5^ ~ Vaccine scheme + COVID-19 + Days from exposure (*N* = 1156)				
Term	Coefficients	Standard error		*P*-value
Intercept	9.5656	0.6366		<0.0001
***Vaccine scheme _(AZ±AZ)_***	**7**.**4257**	**0**.**5327**		**<0**.**0001**
***Vaccine scheme _(Spk±Spk)_***	**8**.**7071**	**0**.**5917**		**<0**.**0001**
***Vaccine scheme _(Spk±Mod)_***	**14**.**2388**	**0**.**6971**		**<0**.**0001**
***Vaccine scheme _(Pfi±Pfi)_***	**17**.**2906**	**1**.**4859**		**<0**.**0001**
**COVID-19**	**5**.**5569**	**0**.**4686**		**<0**.**0001**
**Days from exposure**	**−0**.**0315**	**0**.**0038**		**<0**.**0001**
**Multiple comparisons of means: Tukey's contrasts**				
	Estimate	Standard error	*t* value	*P*-value
**AZ + AZ – Sph + Sph**	**7**.**4258**	**0**.**5328**	**13**.**938**	**<0**.**001**
**Spk + Spk – Sph + Sph**	**8**.**7071**	**0**.**5917**	**14**.**715**	**<0**.**001**
**Spk + Mod – Sph + Sph**	**14**.**2388**	**0**.**6972**	**20**.**423**	**<0**.**001**
**Pfi + Pfi – Sph + Sph**	**17**.**2907**	**1**.**4860**	**11**.**636**	**<0**.**001**
Spk + Spk – AZ + AZ	1.2813	0.5698	2.249	0.147
**Spk + Mod – AZ + AZ**	**6**.**8130**	**0**.**6684**	**10**.**193**	**<0**.**001**
**Pfi + Pfi – AZ + AZ**	**9**.**8649**	**1**.**4598**	**6**.**758**	**<0**.**001**
**Spk + Mod – Spk + Spk**	**5**.**5317**	**0**.**7250**	**7**.**630**	**<0**.**001**
**Pfi + Pfi – Spk + Spk**	**8**.**5836**	**1**.**4962**	**5**.**737**	**<0**.**001**
Pfi + Pfi – Spk + Mod	3.0518	1.5270	1.999	0.246

*Post-hoc* Tukey's tests indicated the significant differences between vaccination schemes. Significant terms are printed in bold and the independent variables of interest are italicised.

Vaccine scheme: AZ + AZ = Astra Zeneca × 2; Spk + Spk = Sputnik V × 2; Spk + Mod = Sputnik V + Moderna; Pfi + Pfi = Pfizer/BioNTech × 2. Reference level: Sph + Sph = Sinopharm × 2.

Antibody levels: titre of anti-SARS-CoV-2 spike protein IgG.

COVID-19: prior diagnosis of COVID-19.

Days from exposure: days elapsed from the last vaccine shot or detected infection.

### Vaccination status and COVID-19 incidence during the Omicron-dominant wave

Of the 514 participants followed up during the wave, 35.8% (184/514) were diagnosed with COVID-19 between 18th December 2022 and 28th February 2022. The incidence in those that had had COVID-19 previously was also high (reinfection rate: 39/121 = 32.2%). The number of vaccine doses received prior to the arrival of Omicron did not appear to have an effect on the COVID-19 infection risk during the wave (Table 4). Participants with two vaccine doses were more likely (~20%) to have COVID-19 during the wave than unvaccinated ones and than individuals with three or more vaccine doses (Table 5). Contact with cases and getting vaccinated during the wave were strong predictors of COVID-19 risk during the wave, and prior COVID-19 was also significantly associated (Table 5).

**Table 4.** COVID-19 attack rate during the Omicron-dominant wave in Santa Fe city, by the number of vaccine doses received prior to the onset of the wave

**Number of vaccine doses**	**0**	**1**	**2**	**≥3**
***N***	41	9	405	59
**Diagnosed with COVID-19**	15	6	146	17
**Attack rate (%)**	36.6	66.7	36.0	28.8

**Table 5.** Logistic regression assessing the association between COVID-19 diagnosis (yes/no) during the Omicron-dominant wave and number of vaccine doses received before the wave, adjusting by age, prior COVID-19, vaccine shot during the wave and number close contacts with cases

Model = COVID-19 (yes/no)~Age + Vaccine doses + Prior COVID-19 + Contact + New vaccine shot (*N* = 484)				
Term	Coefficients (*log odds*)	Odds ratio	Standard error	*P*-value
Intercept	0.4659		0.0723	<0.0001
Age	−0.0010	0.99	0.0012	0.4123
*Vaccine doses (1)*	0.1871	1.21	0.1535	0.2234
***Vaccine doses (2)***	**0.1935**	**1**.**21**	**0**.**0678**	**0**.**0045**
*Vaccine doses (≥3)*	−0.0719	0.93	0.0875	0.4114
**Prior COVID-19**	**−0**.**1045**	**0**.**90**	**0**.**0418**	**0**.**0128**
**Contact**	**0**.**0887**	**1**.**09**	**0**.**0213**	**<0**.**0001**
**New vaccine shot**	**−0**.**5168**	**0**.**59**	**0**.**0390**	**<0**.**0001**

Significant terms are printed in bold and the independent variables of interest are italicised.

Age: in years.

Vaccine doses: number of vaccine shots received prior to the blood sample (4 level variable: 0, 1, 2 and ≥3; reference value = 0).

Prior COVID-19: diagnosis of COVID-19 before the Omicron-dominant wave.

Contact: number close contacts with cases during the Omicron wave.

New vaccine shot: vaccine shot during the Omicron wave.

### Antibody levels and COVID-19 incidence during the Omicron-dominant wave

**Fig. 4.**
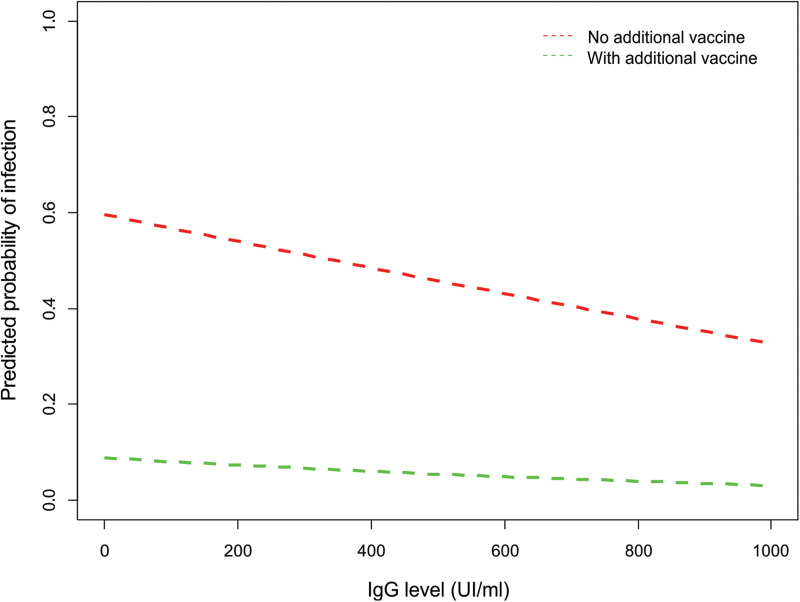
Predicted probability of COVID-19 during the Omicron-dominant wave depending on the levels of antibodies (IgG) against SARS-CoV-2 and the administration of a vaccine dose during the wave. For the simulation contact with cases was set at 1.

There was a strong negative association between antibody levels preceding the Omicron-dominant wave and COVID-19 incidence during the wave (Fig. 4, Table 6 and Table S1 in the Supplementary material). For every 100 UI/ml increase in IgG levels, the risk of infection decreased 12%, adjusting by vaccine shot during the wave and contact with cases (Table 6). Participants with antibody levels >400 UI/ml at the onset of the wave had 67% less chances of being diagnosed with COVID-19 during the wave (Table S1 in the Supplementary material). In addition, receiving a vaccine shot after the onset of the wave and the number of close contacts with cases were strong predictors of COVID-19 risk in all models. A vaccine shot during the wave reduced the probability of COVID-19 by 15-fold (Table 6). Every close contact with a case increased 71% the odds of being diagnosed with COVID-19 during the wave (Table 6).

**Table 6.** Logistic regression assessing the association between COVID-19 diagnosis (yes/no) during the Omicron-dominant wave and preceding antibody levels, adjusting by vaccine shot during the wave and number close contacts with cases

Model = COVID-19 (yes/no) ~ Antibody levels + New vaccine shot + Contact (*N* = 484)				
Term	Coefficients (*log-odds*)	Odds ratio	Standard error	*P*-value
Intercept	0.3857		0.2050	0.0599
***Antibody levels***	**−0.0011**	**0**.**99**	**0**.**0004**	**0**.**0062**
**New vaccine shot**	**−2**.**7105**	**0**.**07**	**0**.**2661**	**<0**.**0001**
**Contact**	**0**.**5349**	**1**.**71**	**0**.**1419**	**0**.**0001**

Significant terms are printed in bold and the independent variable of interest is italicised.

Antibody levels: titre of anti-SARS-CoV-2 spike protein IgG.

New vaccine shot: vaccine shot during the Omicron wave.

Contact: number of close contacts with cases during the Omicron wave.

### Antibody levels and COVID-19 severity and duration

**Fig. 5.**
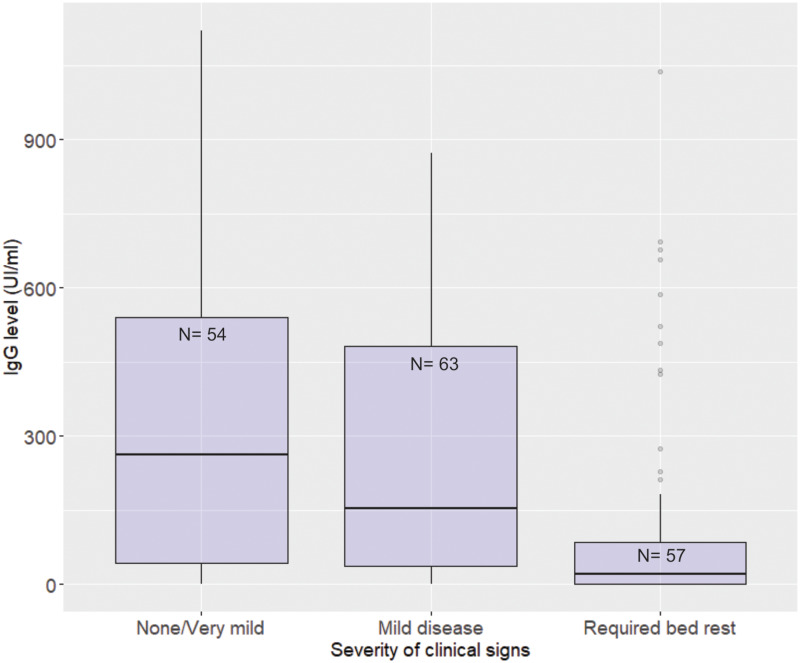
Levels of antibodies (IgG) against SARS-CoV-2 at the onset of the Omicron-dominant wave by the severity of the symptoms when they became infected during the wave.

The ordinal regression model showed that antibody levels were strongly associated with the severity of the symptoms (Table 7, Fig. 5). For every 100 UI/ml increase in the IgG level, the odds of being more likely to have higher disease severity (mild or moderate symptoms *vs*. none or very mild symptoms) decreases 34.8%, holding constant new vaccine shot, age and presence of co-morbidities. The model looking at the association between antibody levels and duration of the symptoms showed a negative trend, but not statistically significant, although borderline (*P* = 0.05; Table S2 in the Supplementary material).

**Table 7.** Ordinal regression model assessing the association between severity of COVID-19 symptoms and antibody levels, adjusting by vaccine shot during the wave, age and co-morbidities

Model = Severity ~ Antibody levels + New vaccine shot + Age + Co-morbidity (*N* = 174)			
Term	Coefficients	Standard error	*P*-value
***Antibody levels***	**−0.0019**	**0.0005**	**<0.0001**
**New vaccine shot**	**−1.0189**	**0**.**4580**	**0**.**0261**
Age	−0.0089	0.0112	0.4235
Co-morbidity	−0.7914	0.4744	0.0953

Significant terms are printed in bold and the independent variable of interest is italicised.

Severity: severity of COVID-19 symptoms; three-level ordinal variable: very mild symptoms, mild symptoms or moderate symptoms.

Antibody levels: titre of anti-SARS-CoV-2 spike protein IgG.

New vaccine shot: vaccine shot during the Omicron wave.

Age: in years.

Co-morbidity: conditions like high blood pressure, diabetes, obesity, heart disease, chronic pulmonary disease, cancer.

## Discussion

The arrival of the variant Omicron was associated with the largest wave of COVID-19 cases in Santa Fe city (Fig. 1), despite immediately prior to the wave a vast majority of the citizens (>90%) had been vaccinated and/or had been infected by SARS-CoV-2. Our characterisation of the acquired humoral defences in Santa Fe prior to the arrival of Omicron showed that a high proportion of the population had immunological memory against COVID-19 (i.e. almost 90% of our sample had detectable antibodies). Moreover, about half of the participants had high antibody levels (>200 UI/ml).

Although the number of cases during the Omicron-dominant wave was much higher than in the previous two waves, the mortality due to COVID-19 was considerably lower. In Santa Fe, before the Omicron-dominant wave, there had been 856 deaths over 55 969 cases (case fatality = 1.5%) and during the wave there were 54 deaths over 36 166 cases (case fatality = 0.15%) (official records of the Ministry of Health of Santa Fe province). This 10-fold lower impact could be attributable to the high level of defences here described, which is supported by the individual-level data presented, in which the severity of COVID-19 was lower in individuals with high preceding antibody levels. However, because there is evidence that suggests that Omicron may be less pathogenic than previous variants [8, 9] it is difficult to infer how much of the reduced severity is due to immunological experience and how much attributable to virus evolution making new variants less pathogenic [10].

A superficial analysis of our data suggests that vaccines did not provide protection during the Omicron wave. In our sample, those who had received two vaccine shots before the wave were 20% more likely to have COVID-19 during the wave than the unvaccinated participants. However, there are two issues that need to be considered before coming to this conclusion. First, because the unvaccinated might be less likely to admit having had COVID-19 or to get tested, there might be more undetected cases among them than in the vaccinated participants, thus leading to a bias that underestimates the incidence in the unvaccinated. Second, the levels of antibodies were found to be highly variable, even within groups with equal number of vaccine doses. Therefore, someone with two vaccine shots could have had little levels of protection at the arrival of Omicron if a long time had passed since the last shot (due to antibody waning; Table 2) and/or because they received a vaccination scheme with poor performance (Table 3). In fact, in our study the strongest predictor of infection risk was getting a vaccine shot during the wave, demonstrating the efficacy of vaccination even in the presence of highly transmissible immune-evading variants. Our results also consistently show that to be protected against such variants the levels defences must be high, which is attained by vaccine boosters.

Antibodies provide protection either through direct obstruction of infection or through their ability to leverage the immune system to eliminate pathogens. The neutralising antibody titres generated in vaccine clinical trials are assumed to be correlated with protective effect and the durability of the protection [11]. Measuring antibody-mediated protection to coronaviruses requires characterisation of immune responses prior to a known exposure or period of risk. Such data are only available from few human challenge experiments, in which volunteers were exposed to experimental infections with human coronaviruses [12]. Some of those studies showed evidence that pre-exposure titres correlated negatively with infection risk and severity [13, 14]. More recent relevant evidence comes from treatments with convalescent plasma. The efficacy of convalescent plasma transfusion as a treatment for COVID-19 was found to depend on the antibody levels of the plasma. The use of plasma with higher anti-SARS-CoV-2 IgG antibody levels was associated with a lower risk of death [15]. In this longitudinal study, we provide individual-level real-world data linking antibody levels and protection against COVID-19 during a period of high risk of exposure to an immune-escaping highly transmissible variant.

The level of antibodies of the participants immediately prior to the arrival of Omicron depended on several factors, of which the most influential ones were prior COVID-19 diagnosis and the days elapsed since last antigen exposure (vaccine shot or infection). It was documented that anti-spike IgG wane quickly [16, 17], and here we confirmed this in a real-world study and showed consequences of waning defences on disease risk and severity.

The number of vaccine doses was only significantly associated with antibody levels when comparing unvaccinated participants with those that received three or more shots. A recent study showed that the neutralisation potency against Omicron was undetectable in sera from most vaccinees, except for individuals recently receiving an RNAm vaccine booster (third dose) [18]. It is noteworthy that in our study this large difference between the titres of two and three shots was maintained when adjusting by days from the last shot.

Another factor that explained the variability in antibody levels among vaccinees was the vaccination scheme. Two inactivated vaccines (Sinopharm) conferred the lowest antibody levels, and schemes that used mRNA platforms (Sputnik + Moderna or Pfizer/BioNTech × 2) the highest titres, whereas both vector vaccines (Astra Zeneca × 2 or Sputnik V × 2) performed between the other two schemes. This is in agreement with what was reported previously [19–21]. Taking into account the above, the highest humoral protection at the time of Omicron arrival was expected in people who had received three or more doses of a vaccination scheme that included mRNA vaccines, who had got the last shot recently and who had had COVID-19 previously.

Prior studies have shown increased antibody evasion and greater breakthrough infection risk of Omicron, compared with previous variants [2, 22, 23]. However, although reduced, the binding of IgG antibodies to the Omicron spike antigen is maintained, and recent data suggest that extra-neutralising antibodies contribute to disease control [24]. This partial immune escape implicates that higher defence levels would be required to reduce the risk and severity of COVID-19 caused by the Omicron variant. Here we present evidence for this in real circumstances. Anti-spike IgG levels and variables that cause antibodies to rise (i.e. prior COVID-19 and a recent boost shot; Table 5) were strong drivers of COVID-19 risk and severity. Our results strongly suggest that to reduce the impact of highly transmissible and immune-escaping variants like Omicron, the acquired defences should be kept high. Therefore, booster vaccine shots during a period of high exposure risk are highly recommended.

The results hereby presented offer an explanation to the epidemiological pattern observed in Santa Fe city during the Omicron-dominant wave. The arrival of the Omicron variant caused the largest COVID-19 epidemic experienced in Santa Fe city since the beginning of the pandemic, but the case fatality observed was 10-fold lower than that of previous waves. The increased number of cases may be have been caused by the immune escape and high transmissibility of Omicron while the existing high immune defences in the population most likely contributed the low impact observed. Disease risk and severity was lowest in individuals with high antibody levels, which highlight the importance of maintaining high defences through vaccination in the presence of immune-escaping variants.

## Data Availability

The datasets used for the statistical analyses of this work are available at https://www.doi.org/10.17632/gpr4fxg44r.1.

## Supplementary material

For supplementary material accompanying this paper visit https://doi.org/10.1017/S0950268822001716. click here to view supplementary material
